# Exosomes in Dogs and Cats: An Innovative Approach to Neoplastic and Non-Neoplastic Diseases

**DOI:** 10.3390/ph14080766

**Published:** 2021-08-04

**Authors:** Emanuela Diomaiuto, Valeria Principe, Adriana De Luca, Flaviana Laperuta, Chiara Alterisio, Antonio Di Loria

**Affiliations:** Department of Veterinary Medicine and Animal Productions, University Federico II of Napoli, Via F. Delpino 1, 80137 Napoli, Italy; emanuela.diomaiuto@alice.it (E.D.); valeria.principe@unina.it (V.P.); adriana.deluca@unina.it (A.D.L.); flaviana.laperuta@unina.it (F.L.); chiara.alterisio@unina.it (C.A.)

**Keywords:** exosomes, extracellular vesicles, miRNA, veterinary medicine, dog, cat, biomarkers

## Abstract

Exosomes are extracellular vesicles with a diameter between 40 and 120 nm, which are derived from all types of cells and released into all biological fluids, such as blood plasma, serum, urine, breast milk, colostrum, and more. They contain proteins, nucleic acids (mRNA, miRNA, other non-coding RNA, and DNA), and lipids. Exosomes represent a potentially accurate footprint of the miRNA profile of the parental cell and can therefore be proposed as potential and sensitive biomarkers, both in diagnosing and monitoring a variety of diseases in humans and animals. Liquid biopsy offers itself as a non-invasive or minimally invasive, pain-free, time-saving alternative to conventional tissue biopsy. Exosomes in both human and veterinary medicine find their major application in neoplastic diseases, but applications in the field of veterinary cardiology, nephrology, reproduction, parasitology, and regenerative medicine are currently being explored. Exosomes can therefore be used as diagnostic, prognostic, and, in some cases, therapeutic tools for several conditions. The aim of this review was to assess the current applications of exosomes in veterinary medicine, particularly in dog and cat patients.

## 1. Introduction

Prokaryotic and eukaryotic cells release extracellular vesicles (EVs): among these are the ectosomes (also recognized as microvesicles or microparticles) and exosomes [1]. Ectosomes pinch off the surface of the plasma membrane via outward budding, while exosomes recognize an endosomal origin. Exosomes are small EVs with a size range of ~40 to 120 nm, and are carriers of bioactive molecules and are secreted by different types of cell after the fusion of multivesicular bodies with their membrane in both physiological and pathological conditions [2].

In 1981, Trams et al. discovered that these micro-vesicles were nothing but exfoliated membrane vesicles containing ecto-enzymes [3], while Pan and Johnstone, in 1983, discovered that sheep reticulocytes, during their maturation process, released these vesicles while eliminating transferrin receptor (Tfr), but they were regarded as cellular waste [4]. Only in 1987 were they given the name “exosomes” [5], but we had to wait until 1996 for their important role to be recognized by Raposo et al.: exosomes are actually mediators of physiological pathways [6]. They are produced by all cell types in physiological and pathological conditions and secreted in all organic fluids, such as blood plasma and serum, urine, breast milk, colostrum, peritoneal fluid, saliva, semen, amniotic fluid, cerebrospinal fluid, bronchoalveolar lavage, bile, synovial fluid, aqueous humors, tears, nasal secretions, pleural effusions, and tumor ascites [7,8,9,10].

As carriers of important messages, exosomes play a crucial role in cell-to-cell communication. Exosomes reach distant target cells, where they release their cargo, represented by proteins, DNA, RNA, microRNAs, cytokines, metabolites, and lipids. The interaction between exosomes and target cells is not yet clear, although the expression of specific molecules on their membrane seems to be fundamental for the binding process [11]. However, in some cases, exosomes release their cargo outside the cell without a direct interaction, probably following possible molecules (e.g., proteins) being able to bind cell receptors [12]. Preliminary data from a recent study also indicated that some exosomes are associated with the surface of red blood cells and that these blood-cell-bound exosomes are also more precise in discriminating cancer patients than cell-free exosomes circulating in the plasma [13].

Exosomes are involved in the immune response, viral pathogenicity, pregnancy, cardiovascular diseases, central nervous system-related diseases, and cancer progression. These characteristics make their application possible in therapeutics and diagnostics. Engineered exosomes are able to deliver therapeutic payloads (short interfering RNA, antisense oligonucleotides, chemotherapeutic agents, and immune modulators), while exosome-based liquid biopsy has shown its utility in diagnosing and issuing a prognosis in patients with cancer and other diseases [14].

Exosome isolation is a current topic of research. Several techniques have been studied, developed, and proposed: ultracentrifugation, ultrafiltration, size exclusion chromatography (SEC), polymer precipitation, immunoaffinity chromatography, and techniques based on microfluids. Each method carries advantages and disadvantages, and may differ in the way the sample is preprocessed and also in the purity and the quality of the exosomes obtained. To evaluate the quality of the harvested exosomes, several methods can be used: electron (SEM and TEM) microscopy, atomic force microscopy (AFM), nanoparticle tracking analysis (NTA), dynamic light scattering coupled with zeta potential determination, Western blotting, fluorescence-activated cell sorting (FACS), and enzyme-linked immunosorbent assays (ELISA) [15,16,17].

## 2. Exosomal Cargo

**Figure 1:**
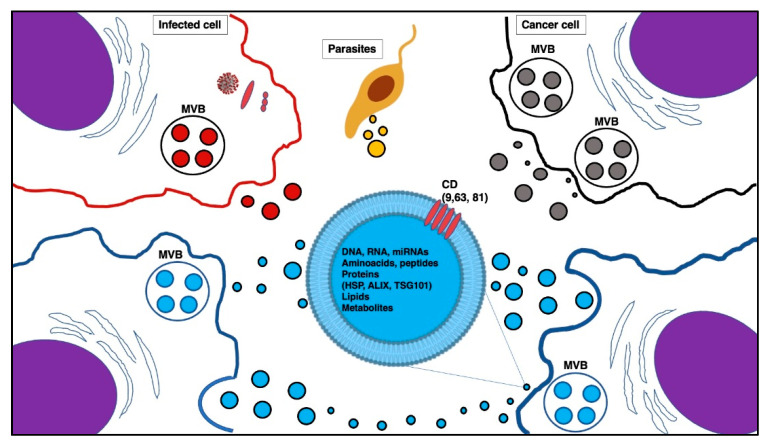
Exosome cross-talking. The biogenesis of exosomes begins with endocytosis to form early endosomes by inward budding, then form late endosomes, and ultimately produce multivesicular bodies (MVBs). MVBs merge with the cell membrane and, by exocytosis, release exosomes into the extracellular environment [1]. Exosomes modulate the recipient cell’s gene expression by initiating cell signaling as well as intercellular transfer of the protein, lipid, and RNA cargo, not only in a functional setting (blue exosomes in the picture) but also in cancer (gray exosomes), infectious (red exosomes), and parasitic (yellow exosomes) diseases, gaining clinical significance because of their potential use as biomarkers or next-generation therapeutics [11]. Abbreviations in the picture: MVB, multivesicular bodies; HSP, heat shock protein; ALIX, ALG-2-interacting protein X; TSG101, tumor susceptibility gene 101; CD, cluster of differentiation.

Exosomes derived from the same parent cell would be expected to contain an analogous protein, nucleic acid, and lipid composition, though it has recently been shown that the molecular composition of exosomes can differ even when the exosomes derive from the same primary cell [1]. These microvesicles contain different biomarkers, proteins, and lipids such as sphingomyelin, cholesterol, phosphatidylserine, and ceramide [18,19,20,21,22,23]. These macromolecules play an important role in various pathological and physiological processes like inflammation, angiogenesis, immune response, cell death, cancer, and neurodegenerative diseases [24]. In Figure 1, we present the structure of exosomes, their content, and the multicellular crosstalk between exosomes and different types of cell.

### 2.1. Proteins

The protein content of exosomes has bben quite well studied. Exosomes are rich in various tetraspanins (CD81, CD82, CD63, CD9) [25,26]. ALIX and TSG101, which are necessary to the formation of multivesicular bodies, are also present. It is also possible to find clathrin, ubiquitin, and lipid raft proteins, as well as targeting adhesion molecules and integrins. The presence of membrane trafficking and fusion proteins is very important for uptake and infusion. In exosomes, it is also possible to find proteins related to the specific parent cell, such as oncogenic proteins or mutant proteins which can be secreted by cancer cells. In exosomes, the endosomal sorting complexes required for transport (ESCRT) play an important role in biogenesis, so they are almost always present in these extracellular vesicles. TSG101 and ALIX are involved in the biogenesis of exosomes as well: in particular, ALIX, TS101, and HSP70 may be used as markers to successfully distinguish exosomes from other extracellular vesicles, even though they are quite recognizable by electron microscopy thanks to their characteristic cup shape [27,28].

Tetraspanins (CD81, CD63, CD9) and flotillin-1 are abundant in the exosomal membranes and may also be considered as markers for their identification.

### 2.2. DNA and RNA

Being secreted by all living cells, exosomes contain biological information and also present specific surface proteins from the parental cells, thus being more representative than cell-free DNA [10]. Recently, Jeppesen et al. published a contradictory paper, which claimed that dsDNA and DNA-binding histones were surprisingly absent in exosomes [29], but not long afterwards, Yokoi et al. re-confirmed via imaging flow cytometry that dsDNA is present in exosomes [30].

In 2006, Ratajczak et al. were the first to identify the presence of messenger RNA in membrane drive cells isolated from embryonic stem cells [31]. In 2007, Valadi et al. demonstrated that exosomes contain RNA, miRNAs, and small non-coding RNA. In this study, the authors proved that these miRNAs have functional activity, giving exosomes an important role as messengers [21].

After the publication of Valadi’s study, in 2012, Bellingham et al. and Nolte-‘t Hoen et al., in two separate studies, used next-generation deep sequencing to profile the RNA associated with extracellular vesicles. Since then, exosomes have been studied as potential biomarkers for various diseases [32,33].

Small non-coding RNAs can be divided into microRNAs, which are between 18 and 25 pairs in length; piwi-interacting RNAs, which are between 26 and 31 base pairs in length; and small nuclear RNAs, which are between 60 and 300 base pairs in length. These types of small non-coding RNA have different cellular functions. Piwi-interacting RNAs are involved in epigenetic modifications, in genome integrity, and in defense transposable elements. MicroRNAs are involved in transcriptional and post-transcriptional regulation and in viral defense [34].

Starting from these functions, it is possible use small non-coding RNA in human and veterinary medicine.

Tumor cells release extracellular vesicles and exosomes as well, and it is possible to find tumor miRNA in these vesicles. For example, through a simple blood sample, it is possible to identify a tumor miRNA coming from any tumor in the body if we can identify the sequences of the specific tumor [35]. Moreover, miRNA can be used to diagnose various diseases.

### 2.3. Lipids

Extracellular vesicles are generally composed of a single phospholipid bilayer, but more layers have been reported. However, all extracellular vesicles, including exosomes, own a membrane structure, so lipids are obligate components of exosomes [36].

The composition of the exosomal membrane is similar to that of the plasma membrane, as they are composed of cholesterol, phospholipids, glycolipids, and integral and peripheral proteins. The major lipids present in the exosomal membrane are phosphatidyl choline, phosphatidyl serine, phosphatidyl ethanolamine, phosphatidyl inositol, sphingomyelin, cerebrosides, gangliosides, and cholesterol [37].

The major differences between the plasma membrane and the vesicle membrane are the symmetric distribution of lipids in the vesicle membrane and the fact that phosphatidyl serine is present on both sides of the vesicle membrane, internal and external. Furthermore, the vesicle membrane has a curve that exposes the hydrophobic core to the aqueous phase. In the exosomes, most lipid classes, including glycosphingolipids, cholesterol, sphingomyelin, and phosphatidyl serine, are expressed more than in the plasma membrane. Additionally, exosomes contain ceramide.

Lipids play an important role in vesicle biogenesis, transport and membrane deformation processes, fission, and fusion [38]. Ceramide is synthesized from sphingomyelin through sphingomyelinase 2 (nMase2) and, in the case of inhibition of this enzyme, proteolipids and proteins released from the exosomes are reduced [39]. Conical lipids such as phosphatidyl ethanolamine and phosphatidyl serine and lysophosphatidylcholine are important for the shape of extracellular vesicles. Cholesterol and sphingomyelin are important for the stabilization of membranes during fusion, while phosphatidyl ethanolamine improves fusion efficiency.

## 3. Exosomes in Neoplastic Diseases

In veterinary medicine, as of today, most studies have focused on the role of exosomes in neoplastic diseases. Exomes indeed mimic the originating cell, representing a fairly accurate footprint of the miRNA profile of the parental cell [40,41], as shown in human lung [42], prostatic [43], colorectal [44], and ovarian cancer [45].

Exosomes influence cancer progression (promoting or inducing neoplasia), paraneoplastic syndromes, and the efficacy of therapy [46]. These vesicles have also been implicated in the angiogenic remodeling of the tumor microenvironment, a critical step in tumor growth and metastatic dissemination [47]. Exosomes promote chemoresistance by enhancing drug resistance properties within cancer cell populations (as seen in human colorectal cancer); chemoresistance is also mediated by horizontal transfer of exosomal miRNAs (as seen in human breast cancer) [48,49].

Opposed to rodents, which are frequently used in experimental studies, companion animals live in the same environment as humans, so their carcinogenesis can be influenced by the same factors: for this reason, malignant canine mammary tumors (CMT) can be considered a study model for breast mammary cancer (BC) in human medicine. Furthermore, exosomes are present in many biological fluids and can be used similarly to minimally invasive liquid biopsies in veterinary medicine [50].

In 2017, Troyer et al. demonstrated that osteosarcoma cells produce exosomes, which have pro-tumorigenic potential in circulating T-cells obtained from healthy dogs. In particular, these exosomes derived from osteosarcoma cells have an immunosuppressive effect on CD4+ and CD8+, reducing their activation and proliferation. Exosomes derived from osteoblasts have an immunosuppression effect, but not exosomes derived from osteosarcoma cells [51].

In 2018, Brady et al. investigated the use of exosomes as biomarkers for osteosarcoma in dogs. They studied the proteomic cargo of exosomes in dogs with osteosarcoma, in dogs with traumatic fractures, and in healthy dogs. The authors demonstrated that it was possible to distinguish dogs with osteosarcoma versus healthy dogs by using an analysis of the proteomic cargo of serum exosomes. Furthermore, this diagnostic approach also proved to be useful to exclude lung metastasis [52].

In 2018, Fish et al. demonstrated that CMT and BC cells released exosomes, and these can be used like biomarkers in dogs and humans. For instance, CMT exosomes, just like BC exosomes, expressed miRNA, especially miR-18a, miR-19a, and miR-181a. The authors demonstrated that these exosomes play a role in cellular proliferation, neoplastic transformation, and tumor progression [50].

In 2020, Howard et al. studied feline mammary tumors, particularly feline mammary adenocarcinoma (FMA), as a model for triple-negative breast cancer (TNBC), a subtype of human breast cancer which does not express estrogen receptors or progesterone receptors, and does not overexpress human epidermal growth factor receptor 2 (HER2). Today, the role of exosomes in TNBC and FMA is not clear, but they can be associated with chemoresistance present in both tumors. However, other studies are needed [53].

In the last few years, studies on lymphoid tumor canine cell lines were published. In 2019, Asada et al. issued a study about the miRNA and protein profile present in exosomes derived from canine lymphoid tumor cell lines. They demonstrated that, despite the major RNA and proteins being similar among all cell lines, the profile of miRNA was different between cell lines. They demonstrated that the presence of some miRNAs, especially miR-151, miR-8908a-3p, and miR-486 and of CD82 protein was different between exosomes derived from vincristine-resistant or vincristine-sensible cell lines [54].

In 2018, Ramos-Zayas et al. published their study about the use of exosomes for cancer immunotherapy. In particular, the authors demonstrated that exosomes derived from venereal tumors can stimulate the immune response in dogs when inoculated intravenously, with therapeutical effects [55].

## 4. Exosomes in Non-Neoplastic Diseases

Though mainly adopted in oncology to detect early malignancy indicators or to monitor metastatic potential, different non-neoplastic diseases may benefit from the developing use of exosome and miRNAs in terms of diagnostics and disease progression monitoring, in both scientific and clinical settings.

### 4.1. Cardiovascular Diseases

Exosomes from various sources have been proven to be cardioprotective. For example, mesenchymal stem cell (MSC)-derived exosomes, when injected into the tail veins of mice undergoing 30 min of myocardial ischemia via ligation of the coronary artery, were able to significantly reduce infarction size after 24 h [56], while plasma-derived exosomes proved to be cardioprotective in an acute setting against ischemia and reperfusion, both in vitro and in vivo, with exosomal HSP70 stimulating TLR4 signaling and leading to the activation of ERK1/2 and p38MAPK, and subsequent HSP27 phosphorylation in cardiomyocytes [57]. This also applied to cardiomyocytes-derived exosomes: when exposed to glucose starvation and co-cultured with endothelial cells, neonatal rat cardiomyocytes shed numerous exosomes containing GLUT1 and GLUT4 transporters, which enabled the uptake of glucose and glycolysis in endothelial cells [58].

Furthermore, various exosome-mediated therapeutic applications have been proposed in a number of cardiovascular models [59]. The majority of the studies investigated the cardioprotective role of different cell-derived exosomes, mostly in myocardial infarction (MI): for example, exosomes from cardiac endothelial cells improved the ejection fraction in rats with MI [60]. Another study showed that exosomes isolated from murine MSCs, when injected into mice with pulmonary arterial hypertension, were able to reverse pulmonary hypertension by modulating pulmonary vascular remodeling [61].

Both in human and veterinary patients, cardiovascular diseases are associated with the dysregulation of miRNAs. A study by Yang and colleagues hypothesized that exosome miRNA could discriminate healthy dogs, dogs with myxomatous mitral valve disease (MMVD), and/or dogs with MMVD and co-existent chronic heart failure (CHF). They attempted to compare the range of miRNA isolated from exosomes with the total plasma miRNA to see if exosomal miRNA was more indicative of the disease. The study evaluated changes in the circulating exosomal miRNA in dogs with MMVD (with and without CHF), comparing them with healthy control dogs, in which a difference between young and old dogs was found. The results showed that cfa-miR-9, cfa-miR-181c, cfa-miR-495, and cfa-miR-599 expression exhibited changes that correlated not only with disease progression and development of heart failure, but also correlated with the dog’s age. This finding could be justified by the prevalence of MMVD in older dogs. This is not the first time that a canine model has supported the evidence of exosomal miRNAs as markers of the different stages of a disease [62].

A 2015 study on mice with doxorubicin-induced dilated cardiomyopathy showed that subjects receiving MSC-derived exosomes showed improved heart function and decreased apoptosis and fibrosis [63]. Beaumier et al. aimed to identify the changes in EV-miRNA expression in dogs receiving doxorubicin (DOX), a chemotherapeutic agent with generally irreversible dose-dependent cardiotoxicity. Changes in the expression of miRNA was then correlated with serum cardiotroponin I (cTnI) measurements and the echocardiographic, electrocardiographic, and histological findings. The results showed that four miRNAs were differentially expressed after the administration of DOX: miR-107, miR-146a, miR-181d, and miR-502. They found a downregulation of miR-107 and miR-146a, as well as an upregulation of miR-502, after just two doses of DOX, as seen in pediatric patients treated with DOX. Specifically, upregulation of miR-502 was detected before any significant cTnI and echocardiographic changes were seen. On the other hand, miR-181d upregulation was only detected in patients with a decreased left ventricle ejection fraction. This particular miRNA inhibited leukemia inhibitory factor (LIF); LIF has cardioprotective effects. In conclusion, EV-miRNAs strongly showed potential as a DOX-induced cardiotoxicity biomarker and should drive the clinician’s choice of treatment, as well as the choice of early cardioprotective strategies during chemotherapy [64].

### 4.2. Reproductive Health

Assisted reproductive techniques (ARTs) are currently available, well-known, and widely used in large animals, whereas in the canine species, the unique features of the reproductive physiology in bitches bring some limitations. The dog is the only mammal in which the ovarian follicles release an immature oocyte in prophase I—requiring 48/72 h to complete maturation in the oviduct—as well as being the only domestic animal in which the follicle undergoes preovulatory luteinization. The environment surrounding the immature oocyte gives it the majority of the sources needed for its metabolism, meaning that valid communication between cells is necessary for correct oocyte development. A high number of proteins in the EVs present in canine oviductal fluid have recently been discovered: the main functions of these EV-related proteins are aiding sperm–oocyte binding, allowing fertilization, and promoting embryo development. The biological function of oviduct-derived exosomes has been studied in pigs, cows, and rats, while in the canine species, we know from previous studies that exosomes play a role in the cumulus-–ocyte interaction. A review by Lee and Saadeldin investigated the properties of canine EVs. Since oocyte maturation happens in the oviduct, the study concluded that EV-related miRNAs are present in the oviductal fluid. The review found the EVs to be potential candidates for regulating oocyte maturation in canine reproduction [65]. To support this hypothesis, a recent study reported that treating cat sperm with oviductal-derived exosomes improved sperm motility [66].

In canine reproduction, semen from male dogs is often cryopreserved to overcome the obstacles to natural breeding in some breeds, live animal transportation and trade. The freezing process, however, can alter the morphology of the sperm, damaging the plasma and acrosomal membranes and resulting in lower post-thaw sperm fertilizing capability. The hypothesis of a study by Qamar was that supplementation with exosomes from canine adipose-derived mesenchymal stem cells (Ad-MSCs) was able to repair the sperm that were damaged in the freezing process. They showed that the supplementation increased the post-thaw mobility and the percentage of live sperm, even if no statistically significant difference was found from the controls. Nonetheless, exosomal-treated sperm had high percentage of intact plasma and acrosome membranes when compared with the controls, as well as significant higher motility and viability [67].

Exosomes have been found in human milk, as well in milk from cows, goats, pigs, buffaloes, pandas, wallabies, horses, camels, and rodents. Among the forementioned species, the most abundant miRNAs have been found in humans, pigs, cows, and pandas, with human milk and cattle milk sharing up to 95% of their miRNA [8,68]. Milk exosomes have been proven to resist the gastrointestinal environment of the newborn, therefore reaching the intestinal cells intact and promoting the immunomodulatory effects and boosting epithelial cell growth of that location, thus stimulating development of the newborn’s gastrointestinal tract [49,69]. Villatoro et al. recently highlighted the abundant presence of exosomes in canine colostrum milk. Exosomes were also shown to be able to interact with mesenchymal stem cells (MSCs), inducing a different secretory profile based on the source of the MSCs: adipose tissue or bone marrow. Exosomes, furthermore, exercised an antioxidant function on canine fibroblasts, which play a part in the maturation of the respiratory system, protecting the newborn from a large number of ROS-mediated pathologies in the early stages of life [70].

### 4.3. Renal Diseases

Urine is a non-invasively accessible biofluid, and renal, bladder, urethral, and prostate diseases, including neoplastic diseases, may benefit from the diagnostic prospect offered by exosomes. Every urothelial cell normally sheds exosomes in the urine, making urinary exosome-derived miRNA a valid tool for monitoring the health of the urinary tract, as it is hypothesized that changes in its physiological condition may be reflected in changes in the miRNA composition of urine [9,71], thus supporting their potential use as early diagnostic biomarkers for kidney diseases. Ichii and colleagues investigated the urinary exosome-derived miRNA profile of kidneys in dogs and cats, and found a total of 277, 276, 295, and 297 miRNAs in the feline renal cortex and medulla, and in the canine renal cortex and medulla, respectively. In veterinary medicine, blood urea nitrogen (BUN) and creatinine (Cr) are universally acknowledged as renal function markers, though they tend to increase when a large part of the kidney is lost and cannot discriminate the injured part of the kidney involved. A previous study from the same group highlighted that the glomerulus tends to be the most commonly afflicted section in canine kidneys, while the tubulointerstitial region is predominantly affected in cats. The authors then focused on identifying novel markers that could function both as disease indicators and therapeutic targets in patients with chronic kidney disease. They performed the study both in dogs and cats. In dogs, miR-26a, miR-146a, miR-486, miR-21a, and miR-10a/b were selected as candidate microRNAs. In particular, urinary exosome-derived miR-26a and miR-10a/b were significantly decreased in dogs with kidney disease, and miR-26a levels had a negative correlation with worsened renal function than other miRNAs. In addition, miR-26a and miR-21a were proven to indicate glomerular and tubulointerstitial damage, respectively. As for cats, let-7b, let-7f, miR-10a, miR-10b, miR-21a, miR-22, miR-26a, miR-27b, miR-146a, miR-181a, miR-191, and miR-486a were identified as biomarker candidates. In particular, let-7b, miR-22, and miR-26a significantly decreased in cats with early kidney disease. Thus, the authors identified that urinary exosome-derived microRNAs in dogs and cats may represent a novel diagnostic tool and their levels could indicate an impairment in renal function, though further studies are necessary for the application of urinary exosome-derived miRNAs to the clinical field [72,73,74].

### 4.4. Parasitic Diseases

During *L. donovani* infection in a murine model, exosomes released by the protozoa, containing the glycoprotein gp63, are responsible for the impairment the immune host response and also reduce the expression of miR-122, a marker for liver impairment. In 2020, Di Loria et al. evaluated the expression of exosomal miR-122, a marker of liver impairment involved in cholesterol metabolism, in leishmaniotic dogs. The authors detected liver dysfunction was associated with a reduction in circulating miR-122 in leishmaniotic dogs, strengthening the hypothesis that *Leishmania* parasites regulate this specific exosomal miRNA through gp63, even in dogs [75].

### 4.5. Orthopaedics and Regenerative Medicine

In veterinary medicine, as opposed to the reality in human medicine, regenerative medicine via the use of mesenchymal stromal cells (MSCs) is a delicate topic. The main therapeutic effect of mesenchymal cell therapy must be attributed to the so-called secretome, a variety of factors secreted by MSCs, mainly composed of cytokines, chemokine, growth factors, and extracellular vesicles. It has been hypothesized that extracellular vesicles alone may be able to heal injuries and tissue damage: this would overcome the practical difficulties we encounter in veterinary medicine regarding the use of stem cell-based protocols. Nonetheless, this approach is recent and still in the early stages of research [76]. El-Tookhy et al. experimentally induced wounds in six dogs and investigated the exosome’s role in wound healing by treating them with exosomal fluid. The results were positive: in dogs treated with exosomes, versus the group treated only with PBS, the healing process was improved both in quality and time, as demonstrated by photographic and histopathological evaluation, thus underlining the efficacy and safety of exosome therapy in wound repair. This may allow veterinarians to prevail over the limitations associated with the use of cell implantation [77]. Exosomes, in addition, are easy to harvest in large quantities and easy to store, particularly through a lyophilization process. EVs are considered a new therapeutic tool particularly in joint and musculoskeletal disorders, such as osteoarthritis, tendon and ligament injuries, and intervertebral disk degeneration [76].

## 5. Conclusions and Future Prospective

The current applications of exosomes in veterinary medicine reviewed in this study are listed in Table 1.

With the evolution of tailored medicine, conventional solid biopsy has been gradually giving way to liquid biopsy, which provides a promising platform for non-invasive diagnosis and prognosis. Undoubtedly, exosomes play an important role in various physiological and pathological processes, and therefore could be potential tools for clinical applications. However, the embryonic development of exosome research, especially in the modern-day veterinary field, still presents some barriers between basic research and clinical practice. Standardization of the classification and extraction methods of exosomes for a minimal concentration of the different body liquids is still needed. Moreover, the simultaneous detection of more molecules carried by exosomes could be able to refine their role as biomarkers in the diagnostic field. Additionally, the biological safety of exosomes must be confirmed before clinical use when these vesicles are used in therapy. The therapeutic potential of exosomes that are tailored to carry other types of cargo (e.g., siRNA) has been investigated recently in experimental animals [78], generating multiple clinical trials registered for diseases such as cancer, wound healing, stroke, and diabetes. In particular, exosomes were obtained from animal cells (mesenchymal stromal cells, dendritic cells, and plasma) and also from plant cells [79]. Research conducted in pigs has shown that the scaling up of exosome production for use in humans is achievable, while lyophilization can be used to preserve exosomal cargo bioactivity. Translational medicine from experimental animals, but also involving companion animals (which frequently represent an animal model for human diseases), is essential for further development in human medicine, but could offer a fast approach to a new exosome-based therapy in veterinary medicine.

**Table 1:** Overview of the current applications of exosomes in veterinary medicine.

Field	Disease or Application	Species	Sample	Isolation	Characterization	Use	Reference
Oncology	Osteosarcoma	Canine	Cell culture	Centrifugation	NTA ^1^	Prognostic	[52]
Oncology	Osteosarcoma	Canine	Serum	Centrifugation	NTA	Diagnostic and prognostic	[53]
Oncology	CMT ^2^	Canine	Cell culture	Centrifugation	TEM ^3^	Diagnostic	[51]
Oncology	FAM ^4^	Feline	/	/	/	Diagnostic	[54]
Oncology	Lymphoid tumor	Canine	Cell culture	Centrifugation	NTA	Diagnostic	[55]
Oncology	Venereal tumor	Canine	Cell culture from biopsies	Centrifugation	TEM	Therapeutic	[56]
Cardiology	MMVD ^5^–CHF ^6^	Canine	Plasma	Sequential ultracentrifugation	Flow cytometry, TEM, NTA	Diagnostic	[63]
Cardiology	Doxorubicin cardiotoxicity	Canine	Plasma	Size-exclusion chromatography	TEM	Diagnostic	[65]
Reproductive health	Semen preservation	Canine	Canine adipose-derived MSCs ^7^	Invitrogen Total Exosome Isolation	TEM	/	[68]
Reproductive health	Evaluation of colostrum quality	Canine	Colostrum	Ultracentrifugation	Western blotting, TEM	/	[72]
Nephrology	CKD ^8^	Canine, feline	Urine	Total Exosome Isolation or miRCURY Exosome Isolation Kit Cells	Immunoblotting, SEM ^9^	Diagnostic	[74,75]
Parasitology	Leishmaniasis	Canine	Serum	ExoQuick	Flow cytometry, (TEM), dynamic light scattering and zeta potential determinations	Prognostic	[77]
Orthopedics and regenerative medicine	Wounds	Canine	MSCs	Ultracentrifugation	Cytofluorimetric analysis	Therapeutic	[79]

^1^ NTA: nanoparticle tracking analysis; ^2^ CMT: canine mammary tumor; ^3^ TEM: transmission electron microscopy; ^4^ FAM: feline mammary adenocarcinoma; ^5^ MMVD: myxomatous mitral valve disease; ^6^ CHF: congestive heart failure; ^7^ MSCs: mesenchymal stem cells; ^8^ CDK: chronic kidney disease; ^9^ SEM: scanning electron microscopy.

## Data Availability

Data sharing not applicable.

## References

[1] Bazzan E., Tinè M., Casara A., Biondini D., Semenzato U., Cocconcelli E., Balestro E., Damin M., Radu C.M., Turato G. (2021). Critical Review of the Evolution of Extracellular Vesicles’ Knowledge: From 1946 to Today. Int. J. Mol. Sci..

[2] Zijlstra A., Di Vizio D. (2018). Size Matters in Nanoscale Communication. Nat. Cell Biol..

[3] Trams E.G., Lauter C.J., Salem N., Heine U. (1981). Exfoliation of Membrane Ecto-Enzymes in the Form of Micro-Vesicles. Biochim. Biophys. Acta (BBA) Biomembr..

[4] Pan B.-T., Johnstone R.M. (1983). Fate of the Transferrin Receptor during Maturation of Sheep Reticulocytes in Vitro: Selective Externalization of the Receptor. Cell.

[5] Johnstone R.M., Adam M., Hammond J.R., Orr L., Turbide C. (1987). Vesicle Formation during Reticulocyte Maturation. Association of Plasma Membrane Activities with Released Vesicles (Exosomes). J. Biol. Chem..

[6] Raposo G., Nijman H.W., Stoorvogel W., Liejendekker R., Harding C.V., Melief C.J., Geuze H.J. (1996). B Lymphocytes Secrete Antigen-Presenting Vesicles. J. Exp. Med..

[7] Tschuschke M., Kocherova I., Bryja A., Mozdziak P., Angelova Volponi A., Janowicz K., Sibiak R., Piotrowska-Kempisty H., Iżycki D., Bukowska D. (2020). Inclusion Biogenesis, Methods of Isolation and Clinical Application of Human Cellular Exosomes. J. Clin. Med..

[8] Cintio M., Polacchini G., Scarsella E., Montanari T., Stefanon B., Colitti M. (2020). MicroRNA Milk Exosomes: From Cellular Regulator to Genomic Marker. Animals.

[9] Weber J.A., Baxter D.H., Zhang S., Huang D.Y., How Huang K., Jen Lee M., Galas D.J., Wang K. (2010). The MicroRNA Spectrum in 12 Body Fluids. Clin. Chem..

[10] Zhou B., Xu K., Zheng X., Chen T., Wang J., Song Y., Shao Y., Zheng S. (2020). Application of exosomes as liquid biopsy in clinical diagnosis. Signal Transduct. Target. Ther..

[11] Heusermann W., Hean J., Trojer D., Steib E., von Bueren S., Graff-Meyer A., Genoud C., Martin K., Pizzato N., Voshol J. (2016). Exosomes Surf on Filopodia to Enter Cells at Endocytic Hot Spots, Traffic within Endosomes, and Are Targeted to the ER. J. Cell Biol..

[12] McGough I.J., Vincent J.-P. (2016). Exosomes in Developmental Signalling. Development.

[13] Tamkovich S., Tutanov O., Efimenko A., Grigor’eva A., Ryabchikova E., Kirushina N., Vlassov V., Tkachuk V., Laktionov P. (2019). Blood Circulating Exosomes Contain Distinguishable Fractions of Free and Cell-Surface-Associated Vesicles. Curr. Mol. Med..

[14] De Toro J., Herschlik L., Waldner C., Mongini C. (2015). Emerging Roles of Exosomes in Normal and Pathological Conditions: New Insights for Diagnosis and Therapeutic Applications. Front. Immunol..

[15] Maisano D., Mimmi S., Russo R., Fioravanti A., Fiume G., Vecchio E., Nisticò N., Quinto I., Iaccino E. (2020). Uncovering the Exosomes Diversity: A Window of Opportunity for Tumor Progression Monitoring. Pharmaceuticals.

[16] Théry C., Witwer K.W., Aikawa E., Alcaraz M.J., Anderson J.D., Andriantsitohaina R., Antoniou A., Arab T., Archer F., Atkin-Smith G.K. (2018). Minimal information for studies of extracellular vesicles 2018 (MISEV2018): A position statement of the International Society for Extracellular Vesicles and update of the MISEV2014 guidelines. J. Extracell. Vesicles.

[17] Willms E., Cabañas C., Mäger I., Wood M.J.A., Vader P. (2018). Extracellular Vesicle Heterogeneity: Subpopulations, Isolation Techniques, and Diverse Functions in Cancer Progression. Front. Immunol..

[18] Raposo G., Stoorvogel W. (2013). Extracellular vesicles: Exosomes, microvesicles, and friends. J. Cell Biol..

[19] Colombo M., Raposo G., Théry C. (2014). Biogenesis, Secretion, and Intercellular Interactions of Exosomes and Other Extracellular Vesicles. Annu. Rev. Cell Dev. Biol..

[20] Möbius W., Van Donselaar E., Ohno-Iwashita Y., Shimada Y., Heijnen H.F.G., Slot J.W., Geuze H.J. (2003). Recycling Compartments and the Internal Vesicles of Multivesicular Bodies Harbor Most of the Cholesterol Found in the Endocytic Pathway. Traffic.

[21] Valadi H., Ekström K., Bossios A., Sjöstrand M., Lee J.J., Lötvall J. (2007). Exosome-mediated transfer of mRNAs and microRNAs is a novel mechanism of genetic exchange between cells. Nat. Cell Biol..

[22] Keerthikumar S., Chisanga D., Ariyaratne D., Al Saffar H., Anand S., Zhao K., Samuel M., Pathan M., Jois M., Chilamkurti N. (2016). ExoCarta: A Web-Based Compendium of Exosomal Cargo. J. Mol. Biol..

[23] Dickens A.M., Tovar-Y-Romo L.B., Yoo S.-W., Trout A.L., Bae M., Kanmogne M., Megra B., Williams D.W., Witwer K.W., Gacias M. (2017). Astrocyte-shed extracellular vesicles regulate the peripheral leukocyte response to inflammatory brain lesions. Sci. Signal..

[24] Howitt J., Hill A.F. (2016). Exosomes in the Pathology of Neurodegenerative Diseases. J. Biol. Chem..

[25] Cocucci E., Racchetti G., Meldolesi J. (2009). Shedding microvesicles: Artefacts no more. Trends Cell Biol..

[26] Andreu Z., Yáñez-Mó M. (2014). Tetraspanins in Extracellular Vesicle Formation and Function. Front. Immunol..

[27] Xu R., Greening D., Zhu H.-J., Takahashi N., Simpson R.J. (2016). Extracellular vesicle isolation and characterization: Toward clinical application. J. Clin. Investig..

[28] Théry C., Ostrowski M., Segura E. (2009). Membrane vesicles as conveyors of immune responses. Nat. Rev. Immunol..

[29] Jeppesen D., Fenix A., Franklin J.L., Higginbotham J.N., Zhang Q., Zimmerman L.J., Liebler D.C., Ping J., Liu Q., Evans R. (2019). Reassessment of Exosome Composition. Cell.

[30] Yokoi A., Villar-Prados A., Oliphint P.A., Zhang J., Song X., De Hoff P., Morey R., Liu J., Roszik J., Clise-Dwyer K. (2019). Mechanisms of nuclear content loading to exosomes. Sci. Adv..

[31] Ratajczak J., Miękus K., Kucia M., Zhang J., Reca R., Dvorak P., Ratajczak M.Z. (2006). Embryonic stem cell-derived microvesicles reprogram hematopoietic progenitors: Evidence for horizontal transfer of mRNA and protein delivery. Leukemia.

[32] Bellingham S.A., Coleman B.M., Hill A.F. (2012). Small RNA deep sequencing reveals a distinct miRNA signature released in exosomes from prion-infected neuronal cells. Nucleic Acids Res..

[33] Nolte-’t Hoen E.N., Buermans H.P.J., Waasdorp M., Stoorvogel W., Wauben M., ’t Hoen P.A. (2012). Deep sequencing of RNA from immune cell-derived vesicles uncovers the selective incorporation of small non-coding RNA biotypes with potential regulatory functions. Nucleic Acids Res..

[34] Esteller M. (2011). Non-coding RNAs in human disease. Nat. Rev. Genet..

[35] Dilsiz N. (2020). Role of exosomes and exosomal microRNAs in cancer. Futur. Sci. OA.

[36] Doyle L.M., Wang M.Z. (2019). Overview of Extracellular Vesicles, Their Origin, Composition, Purpose, and Methods for Exosome Isolation and Analysis. Cells.

[37] Skotland T., Hessvik N.P., Sandvig K., Llorente A. (2019). Exosomal lipid composition and the role of ether lipids and phosphoinositides in exosome biology. J. Lipid Res..

[38] McMahon H.T., Boucrot E. (2015). Membrane curvature at a glance. J. Cell Sci..

[39] Trajkovic K., Hsu C., Chiantia S., Rajendran L., Wenzel D., Wieland F., Schwille P., Brügger B., Simons M. (2008). Ceramide Triggers Budding of Exosome Vesicles into Multivesicular Endosomes. Science.

[40] Vlassov A.V., Magdaleno S., Setterquist R., Conrad R. (2012). Exosomes: Current knowledge of their composition, biological functions, and diagnostic and therapeutic potentials. Biochim. Biophys. Acta (BBA) Gen. Subj..

[41] Bhome R., Del Vecchio F., Lee G.-H., Bullock M.D., Primrose J., Sayan A.E., Mirnezami A.H. (2018). Exosomal microRNAs (exomiRs): Small molecules with a big role in cancer. Cancer Lett..

[42] Rabinowits G., Gerçel-Taylor C., Day J.M., Taylor D.D., Kloecker G.H. (2009). Exosomal MicroRNA: A Diagnostic Marker for Lung Cancer. Clin. Lung Cancer.

[43] Li W., Dong Y., Wang K.J., Deng Z., Zhang W., Shen H.F. (2021). Plasma exosomal miR-125a-5p and miR-141-5p as non-invasive biomarkers for prostate cancer. Neoplasma.

[44] Ogata-Kawata H., Izumiya M., Kurioka D., Honma Y., Yamada Y., Furuta K., Gunji T., Ohta H., Okamoto H., Sonoda H. (2014). Circulating Exosomal microRNAs as Biomarkers of Colon Cancer. PLoS ONE.

[45] Taylor D.D., Gercel-Taylor C. (2008). MicroRNA signatures of tumor-derived exosomes as diagnostic biomarkers of ovarian cancer. Gynecol. Oncol..

[46] Stefanius K., Servage K., Santos M.D.S., Gray H.F., E Toombs J., Chimalapati S., Kim M.S., Malladi V.S., Brekken R., Orth K. (2019). Human pancreatic cancer cell exosomes, but not human normal cell exosomes, act as an initiator in cell transformation. eLife.

[47] Kucharzewska P., Christianson H.C., Welch J.E., Svensson K.J., Fredlund E., Ringnér M., Mörgelin M., Bourseau-Guilmain E., Bengzon J., Belting M. (2013). Exosomes reflect the hypoxic status of glioma cells and mediate hypoxia-dependent activation of vascular cells during tumor development. Proc. Natl. Acad. Sci. USA.

[48] Hu Y., Yan C., Mu L., Huang K., Li X., Tao D., Wu Y., Qin J. (2015). Fibroblast-Derived Exosomes Contribute to Chemoresistance through Priming Cancer Stem Cells in Colorectal Cancer. PLoS ONE.

[49] Chen T., Xie M.-Y., Sun J.-J., Ye R.-S., Cheng X., Sun R.-P., Wei L.-M., Li M., Lin D.-L., Jiang Q.-Y. (2016). Porcine milk-derived exosomes promote proliferation of intestinal epithelial cells. Sci. Rep..

[50] Fish E.J., Irizarry K.J., DeInnocentes P., Ellis C.J., Prasad N., Moss A.G., Bird R.C. (2018). Malignant canine mammary epithelial cells shed exosomes containing differentially expressed microRNA that regulate oncogenic networks. BMC Cancer.

[51] Troyer R.M., Ruby C.E., Goodall C.P., Yang L., Maier C.S., Albarqi H.A., Brady J.V., Bathke K., Taratula O., Mourich D. (2017). Exosomes from Osteosarcoma and normal osteoblast differ in proteomic cargo and immunomodulatory effects on T cells. Exp. Cell Res..

[52] Brady J.V., Troyer R.M., Ramsey S.A., Leeper H., Yang L., Maier C.S., Goodall C.P., Ruby C.E., Albarqi H.A., Taratula O. (2018). A Preliminary Proteomic Investigation of Circulating Exosomes and Discovery of Biomarkers Associated with the Progression of Osteosarcoma in a Clinical Model of Spontaneous Disease. Transl. Oncol..

[53] Howard J., Wyse C., Argyle D., Quinn C., Kelly P., McCann A. (2020). Exosomes as Biomarkers of Human and Feline Mammary Tumours; A Comparative Medicine Approach to Unravelling the Aggressiveness of TNBC. Biochim. Biophys. Acta (BBA) Bioenerg..

[54] Asada H., Tomiyasu H., Uchikai T., Ishihara G., Goto-Koshino Y., Ohno K., Tsujimoto H. (2019). Comprehensive analysis of miRNA and protein profiles within exosomes derived from canine lymphoid tumour cell lines. PLoS ONE.

[55] Zayas Y.R., Franco-Molina M.A., Hernádez-Granados A.J., Triviño D.G.Z., Coronado-Cerda E.E., Mendoza-Gamboa E., Zapata-Benavides P., Ramirez-Romero R., Santana S., Tamez-Guerra R. (2018). Immunotherapy for the treatment of canine transmissible venereal tumor based in dendritic cells pulsed with tumoral exosomes. Immunopharmacol. Immunotoxicol..

[56] Lai R.C., Arslan F., Lee M.M., Sze S.K., Choo A., Chen T.S., Salto-Tellez M., Timmers L., Lee C.N., El Oakley R.M. (2010). Exosome secreted by MSC reduces myocardial ischemia/reperfusion injury. Stem Cell Res..

[57] Vicencio J.M., Yellon D.M., Sivaraman V., Das D., Boi-Doku C., Arjun S., Zheng Y., A Riquelme J., Kearney J., Sharma V. (2015). Plasma Exosomes Protect the Myocardium From Ischemia-Reperfusion Injury. J. Am. Coll. Cardiol..

[58] Garcia N., Oviedo I.O., González-King H., Diez-Juan A., Sepúlveda P. (2015). Glucose Starvation in Cardiomyocytes Enhances Exosome Secretion and Promotes Angiogenesis in Endothelial Cells. PLoS ONE.

[59] Davidson S.M., Takov K., Yellon D. (2016). Exosomes and Cardiovascular Protection. Cardiovasc. Drugs Ther..

[60] Ong S.-G., Lee W.H., Huang M., Dey D., Kodo K., Sanchez-Freire V., Gold J.D., Wu J.C. (2014). Cross Talk of Combined Gene and Cell Therapy in Ischemic Heart Disease. Circulation.

[61] Aliotta J.M., Pereira M., Wen S., Dooner M.S., Del Tatto M., Papa E., Goldberg L.R., Baird G.L., Ventetuolo C., Quesenberry P.J. (2016). Exosomes induce and reverse monocrotaline-induced pulmonary hypertension in mice. Cardiovasc. Res..

[62] Yang V.K., Loughran K.A., Meola D.M., Juhr C.M., Thane K.E., Davis A.M., Hoffman A.M. (2017). Circulating exosome microRNA associated with heart failure secondary to myxomatous mitral valve disease in a naturally occurring canine model. J. Extracell. Vesicles.

[63] Vandergriff A., de Andrade J.B.M., Tang J., Hensley M.T., Piedrahita J.A., Caranasos T.G., Cheng K. (2015). Intravenous Cardiac Stem Cell-Derived Exosomes Ameliorate Cardiac Dysfunction in Doxorubicin Induced Dilated Cardiomyopathy. Stem Cells Int..

[64] Beaumier A., Robinson S.R., Robinson N., Lopez K.E., Meola D.M., Barber L.G., Bulmer B.J., Calvalido J., Rush J.E., Yeri A. (2020). Extracellular vesicular microRNAs as potential biomarker for early detection of doxorubicin-induced cardiotoxicity. J. Veter Intern. Med..

[65] Lee S.H., Saadeldin I.M. (2020). Exosomes as a Potential Tool for Supporting Canine Oocyte Development. Animals.

[66] Ferraz M.D.A.M.M., Carothers A., Dahal R., Noonan M.J., Songsasen N. (2019). Oviductal extracellular vesicles interact with the spermatozoon’s head and mid-piece and improves its motility and fertilizing ability in the domestic cat. Sci. Rep..

[67] Qamar A.Y., Fang X., Kim M.J., Cho J. (2019). Improved Post-Thaw Quality of Canine Semen after Treatment with Exosomes from Conditioned Medium of Adipose-Derived Mesenchymal Stem Cells. Animals.

[68] Van Herwijnen M.J.C., Driedonks T., Snoek B., Kroon A.M.T., Kleinjan M., Jorritsma R., Pieterse C., Hoen E.N.M.N., Wauben M. (2018). Abundantly Present miRNAs in Milk-Derived Extracellular Vesicles Are Conserved Between Mammals. Front. Nutr..

[69] Liao Y., Du X., Yalin L., Lönnerdal B. (2017). Human milk exosomes and their microRNAs survive digestion in vitro and are taken up by human intestinal cells. Mol. Nutr. Food Res..

[70] Villatoro A.J., Martín-Astorga M.D.C., Alcoholado C., Becerra J. (2020). Canine colostrum exosomes: Characterization and influence on the canine mesenchymal stem cell secretory profile and fibroblast anti-oxidative capacity. BMC Vet. Res..

[71] van Balkom B.W., Pisitkun T., Verhaar M., Knepper M.A. (2011). Exosomes and the kidney: Prospects for diagnosis and therapy of renal diseases. Kidney Int..

[72] Ichii O., Ohta H., Horino T., Nakamura T., Hosotani M., Mizoguchi T., Morishita K., Nakamura K., Hoshino Y., Takagi S. (2017). Urinary exosome-derived microRNAs reflecting the changes of renal function and histopathology in dogs. Sci. Rep..

[73] Ichii O., Ohta H., Horino T., Nakamura T., Hosotani M., Mizoguchi T., Morishita K., Nakamura K., Sasaki N., Takiguchi M. (2018). Urinary Exosome-Derived microRNAs Reflecting the Changes in Renal Function in Cats. Front. Vet. Sci..

[74] Ichii O., Otsuka S., Ohta H., Yabuki A., Horino T., Kon Y. (2014). MicroRNA expression profiling of cat and dog kidneys. Res. Vet. Sci..

[75] Di Loria A., Dattilo V., Santoro D., Guccione J., De Luca A., Ciaramella P., Pirozzi M., Iaccino E. (2020). Expression of Serum Exosomal miRNA 122 and Lipoprotein Levels in Dogs Naturally Infected by Leishmania infantum: A Preliminary Study. Animals.

[76] Mocchi M., Dotti S., Del Bue M., Villa R., Bari E., Perteghella S., Torre M.L., Grolli S. (2020). Veterinary Regenerative Medicine for Musculoskeletal Disorders: Can Mesenchymal Stem/Stromal Cells and Their Secretome Be the New Frontier?. Cells.

[77] El-Tookhy O.S., Shamaa A.A., Shehab G.G., Abdallah A.N., Azzam O.M. (2017). Histological Evaluation of Experimentally Induced Critical Size Defect Skin Wounds Using Exosomal Solution of Mesenchymal Stem Cells Derived Microvesicles. Int. J. Stem Cells.

[78] Alvarez L., Seow Y., Yin H., Betts C., Lakhal S., A Wood M.J. (2011). Delivery of siRNA to the mouse brain by systemic injection of targeted exosomes. Nat. Biotechnol..

[79] US National Library of Medicine Clinical Trials. https://clinicaltrials.gov/.

